# Systems pharmacogenomics identifies novel targets and clinically actionable therapeutics for medulloblastoma

**DOI:** 10.1186/s13073-021-00920-z

**Published:** 2021-06-21

**Authors:** Laura A. Genovesi, Amanda Millar, Elissa Tolson, Matthew Singleton, Emily Hassall, Marija Kojic, Caterina Brighi, Emily Girard, Clara Andradas, Mani Kuchibhotla, Dharmesh D. Bhuva, Raelene Endersby, Nicholas G. Gottardo, Anne Bernard, Christelle Adolphe, James M. Olson, Michael D. Taylor, Melissa J. Davis, Brandon J. Wainwright

**Affiliations:** 1 The University of Queensland Diamantina Institute, The University of Queensland, Woolloongabba, QLD 4102 Australia; 2 Institute for Molecular Bioscience, The University of Queensland, St Lucia, QLD 4072 Australia; 3 ARC Centre of Excellence for Convergent Bio-Nano Science and Technology, The University of Queensland, St Lucia, QLD 4072 Australia; 4 Australian Institute for Bioengineering and Nanotechnology, The University of Queensland, St Lucia, QLD 4072 Australia; 5 Clinical Research Division, Fred Hutchinson Cancer Research Center, Seattle, WA 98109 USA; 6 Brain Tumour Research Program, Telethon Kids Cancer Centre, Telethon Kids Institute, Nedlands, WA 6009 Australia; 7 Bioinformatics Division, The Walter and Eliza Hall Institute of Medical Research, Parkville, Victoria 3052 Australia; 8 Department of Medical Biology, Faculty of Medicine, Dentistry and Health Sciences, The University of Melbourne, Melbourne, Victoria 3010 Australia; 9 QFAB Bioinformatics, Institute for Molecular Bioscience, The University of Queensland, St Lucia, QLD 4072 Australia; 10 Programme in Developmental and Stem Cell Biology, Arthur and Sonia Labatt Brain Tumour Research Centre, Hospital for Sick Children, Toronto, Ontario MSG 1X8 Canada; 11 Division of Neurosurgery, Hospital for Sick Children, Toronto, Ontario MSG 1X8 Canada; 12 Department of Clinical Pathology, Faculty of Medicine, Dentistry and Health Sciences, The University of Melbourne, Melbourne, Victoria 3010 Australia

**Keywords:** Medulloblastoma, Genetic screen, Protein interaction network, Drug target, Microtubule stabilization

## Abstract

**Background:**

Medulloblastoma (MB) is the most common malignant paediatric brain tumour and a leading cause of cancer-related mortality and morbidity. Existing treatment protocols are aggressive in nature resulting in significant neurological, intellectual and physical disabilities for the children undergoing treatment. Thus, there is an urgent need for improved, targeted therapies that minimize these harmful side effects.

**Methods:**

We identified candidate drugs for MB using a network-based systems-pharmacogenomics approach: based on results from a functional genomics screen, we identified a network of interactions implicated in human MB growth regulation. We then integrated drugs and their known mechanisms of action, along with gene expression data from a large collection of medulloblastoma patients to identify drugs with potential to treat MB.

**Results:**

Our analyses identified drugs targeting CDK4, CDK6 and AURKA as strong candidates for MB; all of these genes are well validated as drug targets in other tumour types. We also identified non-WNT MB as a novel indication for drugs targeting TUBB, CAD, SNRPA, SLC1A5, PTPRS, P4HB and CHEK2. Based upon these analyses, we subsequently demonstrated that one of these drugs, the new microtubule stabilizing agent, ixabepilone, blocked tumour growth in vivo in mice bearing patient-derived xenograft tumours of the Sonic Hedgehog and Group 3 subtype, providing the first demonstration of its efficacy in MB.

**Conclusions:**

Our findings confirm that this data-driven systems pharmacogenomics strategy is a powerful approach for the discovery and validation of novel therapeutic candidates relevant to MB treatment, and along with data validating ixabepilone in PDX models of the two most aggressive subtypes of medulloblastoma, we present the network analysis framework as a resource for the field.

**Supplementary Information:**

The online version contains supplementary material available at 10.1186/s13073-021-00920-z.

## Background

Medulloblastoma (MB) is the most common malignant paediatric brain tumour accounting for 20–25% of all childhood brain cancers [1]. Remarkable progress in defining the molecular and cytogenetic features of MB has confirmed the biological heterogeneity of MB, with several studies defining at least four molecular subgroups: Wingless (WNT), Sonic Hedgehog (SHH), Group 3 (Gp3) and Group 4 (Gp4) and up to 12 subtypes within the subgroups [2–7]. Current treatment options including surgical resection, craniospinal irradiation and high-dose chemotherapy have improved survival rates to approximately 70–75% for children with average-risk MB [8–11]. The outcome for high-risk patients however remains poor and survivors frequently relapse and face significant long-term neurocognitive, endocrine and physical sequelae as a consequence of aggressive treatment protocols. Several studies have demonstrated that the stratification of patients on the basis of the distinct molecular features defining MB subgroups can have a profound impact on their clinical outcome (as reviewed in [12]), warranting the integration of subgroup-specific molecular profiles into the stratification of patients and ultimately, the designation of treatment protocols. A multicentre clinical trial investigating the stratification of patients on the basis of molecular subgroup (WNT, SHH or non-WNT/ non-SHH) followed by clinical risk (low, average or high-risk) (SJMB12) is currently underway. This trial will evaluate whether the addition of the SHH pathway Smoothened (SMO) antagonist, vismodegib, and new chemotherapeutic agents, gemcitabine and pemetrexed, to standard chemotherapy will improve treatment outcome for patients with SHH MB and non-WNT/non-SHH MB respectively. In parallel to this study, the identification and development of additional novel targeted therapeutic options aimed towards the specific molecular drivers of MB is urgently required. Ultimately, this will enable a new era in the treatment of MB, where patients are preselected for a particular targeted therapy based on molecular phenotype, an approach that will improve clinical benefit and may lead to a reduction in the toxicity associated with existing non-precision treatments.

Systems pharmacogenomics, an approach that integrates computational modelling with biomedical, biomolecular and genomic data, has the potential to make a substantial contribution to the development of novel therapeutics or to the repurposing of existing drugs for new diseases. Whilst pharmacogenomics sets out a framework for identifying the relationship between the genome and drug response, systems pharmacogenomics places this work in the context of systems biology. By identifying how the network of interacting genes and proteins cooperate to dictate drug response, we can use patterns in the network to target therapy. Pharmacogenomics approaches have shown benefit in other cancers [13, 14]; however, the scarcity of actionable mutations in MB has historically limited the application of genomics for drug selection [15]. The development of a computational strategy for prioritizing candidate drugs represents an alternative approach to rapidly evaluate existing therapeutics against a computational model prior to moving to experimental work.

Here, we present a systems approach to the identification of novel drug targets for all non-WNT MB, using a protein-protein interaction network constructed from genes identified as promoting aggressive disease in our transposon-driven mouse model of MB and experimentally verified, literature curated protein-protein interactions (PPI) [16]. Briefly, *Sleeping Beauty* (*SB*)-induced mutagenesis in the *Patched 1* (*Ptch1)* heterozygous mouse model resulted in accelerated MB tumorigenesis, with transposon common insertion sites (CIS) determined to identify candidate causative candidate cancer genes driving accelerated MB development [16]. The local protein network for each CIS-derived candidate cancer gene was generated from experimentally determined PPI data and these local protein networks were integrated to generate a protein interaction network comprising the CISs and their interacting proteins. Unexpectedly, the CIS-derived candidate cancer genes and associated protein network was capable of distinguishing the molecular subgroups of human MB, indicating that the *SB* mouse model of MB captured the genetic diversity and common pathways underpinning distinctive human MB subgroups [16]. Given the power of this integrated computational and experimental approach to predict the complex biology underlying MB, here we have used this functionally defined PPI network to define novel therapeutic approaches for all molecular subgroups of human MB.

We restricted this analysis to non-WNT MB since the WNT subgroup is associated with greater than 95% long-term survival and is by some margin the least frequent subgroup. We chose to focus on over-expressed genes in human MB, given that majority of drugs are inhibitors and block protein function. Additionally, elevated mRNA expression has been identified as a strong characteristic hallmark in the computational identification of novel anti-cancer drug targets using high-throughput data [17]. Working within the drug-repurposing paradigm, we created a drug-target network using the DrugBank database and significantly over-expressed genes identified from human MB expression data (Additional file 1: Fig. S1). We then identified druggable targets, defined exclusively as proteins with validated drug interactions rather than proteins with predicted drug interactions. Additionally, we focused on protein network/drug combinations that were in common between SHH, Gp3 and Gp4 MB on the basis that an ideal therapeutic would target all three subgroups. Such therapeutics are likely to have the greatest clinical impact with, ultimately, a simplified clinical trial design afforded by targeting three tumour subgroups simultaneously.

Several of the targets we predicted by this approach, including Aurora kinase A (AURKA), cyclin-dependent kinase 6 (CDK6), cyclin-dependent kinase 4 (CDK4) and checkpoint kinase 2 (CHEK2), are validated targets in MB and currently have drugs targeting them under development as MB therapeutics [18–21], lending weight to the novel predictions that also arose from our analysis. This principled and data-driven systems pharmacology approach not only identified new and existing protein targets but also identified a network of therapeutics that would potentially target those proteins. Here, one such therapeutic, ixabepilone, targeting the functional hub tubulin beta chain (TUBB) defined in our analyses was tested in Gp3 and SHH patient-derived xenograft (PDX) models leading to the identification of considerable anti-tumour activity in all cases. Our experimental data in Gp3 and SHH PDX MB coupled with the success of many network leads as drug targets validates the capability of this drug-target network to identify common therapeutic strategies for multiple subgroups of MB.

## Methods

### Bioinformatics and statistics

*SB*-mutagenesis CIS-derived candidate cancer genes were identified and mapped to mouse and human protein coding genes as described in our previous study [16]. Local protein interaction networks for each *SB*-mutagenesis CIS-derived candidate cancer gene were constructed to generate a protein interaction network consisting of 622 proteins and 666 edges as previously described [16]. Cytoscape 3.2.0 was employed to analyse and visualize the network [22]. Human primary MB (*n* = 285) were profiled on the Affymetrix Human Exon 1.1ST 24-Array platform at the German Cancer Research Center (DKFZ, www.dkfz.de; Heidelberg, Germany, available through the Gene Expression Ombibus: GSE37382). Sample annotation was collected from the GSE37382 series matrix file. Expression data were log_2_transformed and *z*-score normalized using R. Differential expression was calculated using the LIMMA package in Bioconductor. A threshold of 0.05 was applied to the FDR corrected *P* value to identify significantly differentially expressed genes relative to normal foetal and adult cerebellum. Normal foetal and adult cerebellum samples (GSE167447) were processed in the same way. All data were then mean centred and scaled to ensure comparable distributions prior to differential expression analysis.

Probes corresponding to the proteins residing in our interaction network were selected based on the probe annotation file for GPL11532, and expression data were extracted from the GSE37382 Series Matrix file. No additional probe selection was applied, and if more than one probe corresponded to a listed gene, all probes for that gene were selected. Expression data were imported into our protein interaction network to identify over-expressed genes present in the network. Drugs and their molecular targets were obtained from DrugBank version 4.3 [23] and integrated with the protein interaction network to create a drug-target protein interaction network consisting of 1274 nodes and 1435 edges. Briefly, human protein-protein interaction data was collected from International Molecular Exchange (IMEX) Consortium partner databases [24]. Hits from the mutagenesis screen were mapped to human orthologs, and protein interactions involving those orthologs were retrieved and used to construct the base protein interaction network. We then retrieved drugs known to target proteins in this network from Drug Bank and added drug-protein interactions to the base protein interaction network. This protein-protein-drug network was used for all subsequent analyses.

For subgroup-specific analysis, a refined protein interaction network was created including only the significantly over-expressed proteins (*FDR P* < 0.05) and their direct protein-protein interactors. The number of patient samples, significantly deregulated proteins, putative drug targets, putative drugs and the nodes and edges contained in each of the subgroup-specific networks is included in Additional file 2: Tables S1 and Additional file 3: Table S2. Drugs and their molecular targets were mapped to proteins residing in this over-expressed network, creating drug-target networks specific to each MB subgroup, and to identify significantly over-expressed druggable proteins specific to each individual subgroup of MB. Differential expression data was integrated with the base network, with the results (fold change and p value) of all four comparisons added to protein nodes as node attributes. For each comparison, we then filtered nodes to select those that were significantly upregulated and then generated a sub-network that included the direct neighbours of these upregulated nodes (that is, we selected additional nodes one step out from the upregulated protein). This expansion step pulled in both neighbouring proteins, and any drugs targeting the upregulated proteins, from the base network described above. Expression levels of tubulin beta class 1 (*TUBB)* in MB PDXs were obtained from microarray analysis results from the R2 Genomics Analysis and Visualization Platform (http://r2.amc.nl) using the Mixed Paediatric PDX (public) dataset. *TUBB* probesets in each database with the highest average signals were selected for analysis.

### Human cohorts

Human gene expression data for medulloblastoma samples (GSE37382) and normal foetal and adult cerebellum samples (GSE167447) were downloaded from the Gene expression Omnibus and processed as described above. All data are publicly available: see the “Availability of data and materials” section for details.

### Mice

Six to ten-week-old NOD.Cg-*Prkdc*^scid^ *Il2rg*^*tm1Wjl*^*/SzJ* (NSG) mice were originally purchased from The Jackson Laboratory (Bar Harbor, ME) and maintained as a colony at the Translational Research Institute, University of Queensland. Female athymic nude mice (Hsd:Athymic Nude-*Foxn1*^*nu*^) at 4 to 6 weeks of age and approximately 20 g were purchased from Envigo (Kent, WA) or the Animal Resources Centre (Perth, Australia). Animals were housed in a barrier facility with food and water provided ad libitum on a 12-h light/dark cycle. All applicable international, national, and/or institutional guidelines for the care and use of animals were followed. All experiments were performed with approvals from either The Fred Hutchinson Cancer Research Centre Institutional Animal Care and Use Committee, Telethon Kids Institute Animal Ethics Committee or The University of Queensland Molecular Biosciences committee (IMB/386/18).

### Medulloblastoma PDX mouse models

Studies were conducted using four PDX MB models, Med-211FH, Med-411FH and Med-1712FH and BT084. Med-211FH, Med-411FH and Med-1712FH were generated in the Olson laboratory (Fred Hutchinson Cancer Research Center, Seattle) using paediatric patient tumour tissue obtained from Seattle Children’s Hospital with approval from the Institutional Review Board. Med-211FH, Med-411FH and Med-1712FH are publicly available from https://research.fredhutch.org/olson/en/btrl.html, with details of these models published [25]. Med-211FH is a *MYC*-amplified Gp3 MB with classic morphology that was derived from a 2.8-year-old male patient. Med-411FH is a *MYC*-amplified Gp3 MB with large cell/anaplastic morphology that was derived from a 3-year-old male patient. Med-1712FH is a SHH MB with desmoplastic/nodular morphology, derived from a 4.9-year-old patient. BT084 PDX model represents SHH MB harbouring *TP53* mutation and was gifted from Stefan Pfister (DKFZ, [unpublished]). BT084 cells were modified by lentiviral infection to drive expression of luciferase and green fluorescent protein (GFP) using the pCL20-MSCV-GFP-ires-Luc2 construct kindly provided by Dr Richard Williams of St Jude Children’s Research Hospital (Memphis, USA). PDX lines were generated by implanting tumour cells directly into the cerebellum of immunocompromised mice (NSG or athymic nude) within hours of surgical removal from the patient, and then propagating them from mouse to mouse exclusively without in vitro passaging as previously described [25]. Xenografted tumours were subjected to genomic analysis and compared to the primary tumour from which they originated [25].

### Subcutaneous and orthotopic xenografts

To generate subcutaneous tumours, tissue was harvested from intracranial tumours in symptomatic donor mice and immediately processed for transplant into the subcutaneous space of the flank in NSG mice. Tissue was processed in serum-free Dulbecco’s Modified Eagle’s Medium (DMEM) or phosphate buffered saline (PBS) by trituration through an 18-g or 21-g needle to generate a single cell suspension. The suspension was filtered, centrifuged and re-suspended in 1:1 serum-free DMEM:Matrigel solution to a concentration of 15,000/μl. One hundred microliters of the tumour cell suspension (1.5 × 10^6^ cells) was injected into the subcutaneous space of the flank of NSG mice.

For orthotopic xenografts, athymic nude mice or NSG were anesthetized and a small incision was made in the skin to expose the skull. A handheld microdrill with a 0.7-mm or 0.9-mm burr was used to create a hole in the calvarium situated above the right cerebellar hemisphere, 2 mm lateral (right) to the sagittal suture, 2 mm posterior of the lambdoid suture. Two microliters of cell suspension (100,000 cells) in either DMEM (Med-211FH and Med411-FH) or Matrigel (BT084) was injected into the brain parenchyma approximately 2 mm under the dura. SurgiFoam was inserted into the burr hole site. The incision was closed with tissue glue.

### Systemic chemotherapy

Ixabepilone (BMS-247550, Selleckchem) was used for all experiments. Ixabepilone was dissolved in 2% DMSO, 30% PEG300, 2% Tween-80 and administered by intravenous injection every 4 days for three doses (q4d3) for a total of two cycles, with 2 weeks off in between cycles.

### In vivo efficacy studies—subcutaneous tumours

Studies were conducted using Med-211FH, Med-411FH and Med-1712FH subcutaneous tumours between 200 and 500 mm^3^ in size. Tumour volume measurements were taken biweekly and mouse weights measured daily. Tumours were measured with calipers, and tumour volume was calculated using the equation: ((length × width^2^)/2). Mice were stratified into groups according to tumour size and the groups were randomly assigned by rolling enrolment to therapeutic treatment. Treatment was administered for 30 days or until tumour size reached maximum size requiring ethical collection. Changes in tumour volume for each treatment group were reported based upon tumour measurements on day 30 relative to day 1 of treatment. Tumour measurements from mice bearing Med-211FH, Med-411FH and Med-1712FH PDX flank tumours treated with ixabepilone or vehicle were analysed using linear mixed models analysis in R. A subset of additional tumour bearing mice were established as described above for assessing the mechanism of tumour regression in response to ixabepilone. For this analysis, drug-treated tumours were collected approximately 5–10 h after the second dose of ixabepilone whereby tumours had demonstrated evidence of regression and compared to matched vehicle-treated mice at the same timepoint. Tumours were preserved by paraformaldehyde fixation and processed for immunoanalysis.

### In vivo efficacy studies—orthotopic tumours

Orthotopic xenotransplantation was performed in athymic nude mice for Med-211FH and BT084 or NSG mice for Med-411FH. For Med-411FH, tumour growth was confirmed in each mouse by the onset of symptoms and randomly assigned into treatment groups. For Med-211, tumour growth was confirmed by the onset of symptoms and the cohort of mice were stratified according to the severity of symptoms and randomly assigned into treatment groups. For BT084, tumour growth was monitored weekly by bioluminescence using a Lago X small animal imager (Spectral Instruments Imaging, USA) and treatment commenced once bioluminescence reached approximately 5 × 10^5^ photons/second. Mice enrolled in the study were observed daily and weights recorded daily (Med-411FH) or three times per week (Med-211FH and BT084). Euthanasia was performed prior to the end of the study if mice demonstrated signs of tumour-related morbidity or lost more than 20% body weight loss. Kaplan-Meier survival estimates were calculated using GraphPad Prism 7.0a software, and survival curves are presented in results. Differences in survival between groups were performed using Log rank (Mantel Cox) test. Median survival and 95% confidence interval (95% CI) are reported.

### Immunoblotting analysis of PDX tumours

Whole cell extracts from MB PDX were prepared using radioimmunoprecipitation assay buffer (RIPA) buffer (150 mM NaCl, 1% Nonidet P-40, 0.5% sodium deoxycholate, 0.1% SDS, 25 mM Tris pH 7.4) with protease and phosphatase inhibitors (Cell Signalling, 5872). Total protein concentrations were determined using the BCA kit (Pierce, 23225). Equal amounts of total protein extract were resolved on SDS-PAGE and transferred to a polyvinylidene difluoride membrane using a transfer apparatus according to manufacturer’s protocols (Invitrogen, B1000). After incubation with 5% non-fat milk in TBST (10 mM Tris, pH 7.6, 150 mM NaCl, 0.1% Tween 20) for 60 min, the membrane was washed once with TBST and incubated with antibodies against: TUBB3 (Covance, MRB-435P, 1:1000) and Beta-actin (BACT) (abcam, ab8224, 1:500) at 4° for 16–18 h. Membranes were washed three times for five minutes and incubated with a 1:2500 of horseradish peroxidase-conjugated anti-mouse or anti-rabbit secondary antibody (1:2500) for 1 h at room temperature. Blots were washed with TBST three times and developed using the ECL system (Amersham Biosciences) according to manufacturer’s protocol.

### Immunofluorescence analysis of PDX tumours

Antibody markers were analysed on seven micron, paraffin-embedded sections via standard immunofluorescence techniques using the following antibodies: Phospho-Histone H3 (pHH3) (Cell Signalling #9701, 1:100), marker of proliferation (Ki67) (BD Pharmingen #556003, 2.5 μg/ml) and cleaved caspase-3 (CC3) (abcam #ab2302, 10 μg/ml). High-temperature unmasking was performed with preheated pH 6.0 citrate buffer (Vector Labs) for 5 min, and mouse-on-mouse blocking reagent was used to block non-specific binding of mouse primary antibodies (Vector Labs). For the quantitation of positively staining pHH3, CC3 and/or Ki67 cells: tumour cell nuclei as identified by DAPI were scored for the presence or absence of pHH3, CC3 and Ki67 staining in five fields of view per tumour in triplicate for each treatment group [26]. GraphPad Prism 7.0a software was used to analyse immunofluorescence difference between treatment groups using a Welch’s t test for pHH3 and a Holm-Sidak corrected multiple t test for the CC3 and Ki67 co-stain.

## Results

### Regulatory networks identified in a MB mouse model discover novel drug candidates common to all human non-WNT MB

**Fig. 1:**
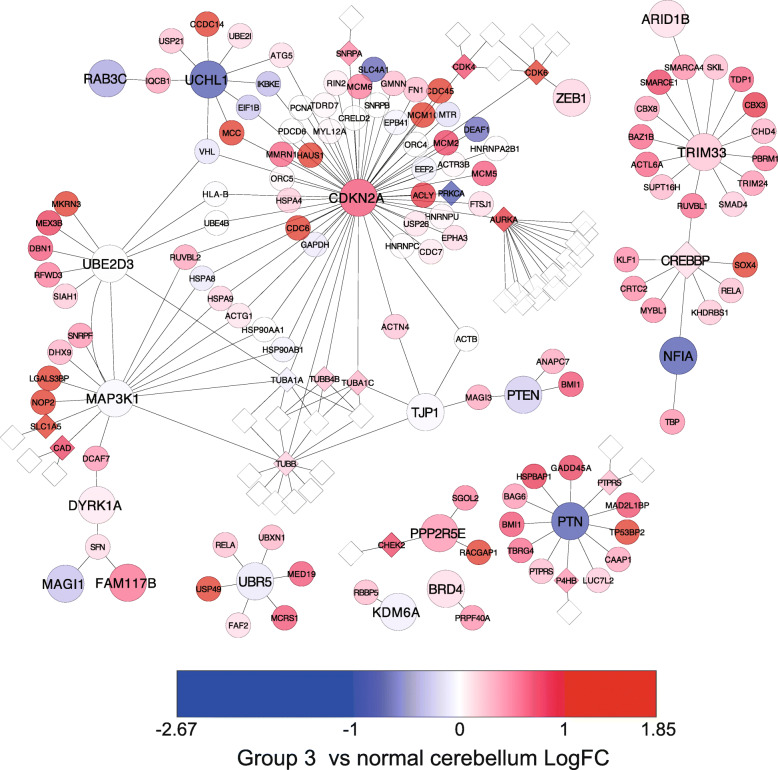
Local PPI network representing the druggable proteins for non-WNT MB. CIS-derived candidate cancer genes are represented by large circles, whilst small circles represent the proteins that interact with these CIS-derived candidates. Nodes coloured in red–light red represent significantly over-expressed proteins in Gp3 MB, whilst blue–light blue nodes represent significantly under-expressed proteins in Gp3 MB. White small diamond shaped nodes represent drugs for that protein. For expression data for each node in the network, see Additional file 4

To discover novel drugs with potential anti-tumour activity in human MB, we identified differentially expressed genes common to all non-WNT MB (*P* < 0.05), namely SHH, Gp3 and Gp4 MB, and mapped expression values to the functionally derived protein interaction network we have described previously [16]. Of the 168 significantly differentially expressed genes, 84 were significantly over-expressed in all three MB subgroups, SHH, Gp3 and Gp4, and are subsequently referred to here as the commonly over-expressed genes for non-WNT MB. A refined PPI network was then created including only the proteins from these commonly over-expressed genes and their direct interaction partners (Fig. 1, Additional file 4: Table S3). To identify the druggable components of this network, drugs with known protein targets were mapped to the network, generating a drug-target network containing 145 proteins and 36 drugs. The network contains one large connected component, with several smaller components disconnected from the main part of the network (Fig. 1). The central component of the network contains proteins derived from multiple genes that we had previously identified through our functional screen as drivers of MB growth: cyclin-dependent kinase inhibitor 2A (CDKN2A), zinc finger E-Box binding homeobox 1 (ZEB1), tight junction protein 1 (TJP1), mitogen-activated protein kinase kinase kinase 1 (MAP3K1), family with sequence similarity 117, member B (FAM117B), dual-specificity tyrosine-(Y)-phosphorylation regulated kinase 1A (DYRK1A), ubiquitin-conjugating enzyme E2D 3 (UBE2D3), ubiquitin C-terminal hydrolase L1 (UCHL1), member RAS oncogene family (RAB3C), phosphatase and tensin homologue (PTEN), membrane-associated guanylate kinase, WW and PDZ domain (MAGI1) and ubiquitin protein ligase E3 component N-recognin 5 (UBR5) [16]. Seven over-expressed genes identified as druggable interact with the CIS-derived candidate cancer proteins residing in this central component of the network (Table 1). Five of these, AURKA, CDK4, CDK6, small nuclear ribonucleoprotein polypeptide A (SNRPA) and TUBB, directly interacted with CIS CDKN2A, suggesting the therapeutic targeting of any of these druggable proteins may influence functions associated with CDKN2A. The MAP3K1 hub contains three significantly over-expressed proteins that are druggable: TUBB, carbamoyl-phosphate synthetase 2, aspartate transcarbamylase, and dihydroorotase (CAD) and solute carrier family 1 (neutral amino acid transporter) member 5 (SLC1A5). The identification of multiple significantly over-expressed druggable proteins interacting with CDKN2A and MAP3K1 further emphasizes the role for both of these proteins not only in driving accelerated MB tumorigenesis [16], but also as potential foci of targeted therapies.

**Table 1:** Significantly over-expressed druggable network proteins and their associated CISs common to all non-WNT MB

Network protein	CIS	Evidence as a novel therapeutic target for MB
AURKA	CDKN2A	[1, 2]
CAD	MAP3K1	
CDK4	CDKN2A	[3–6, 8]
CDK6	CDKN2A/ ZEB1	[3–6, 8]
CHEK2	PPP2R5E	[9]
P4HB	PTN	
PTPRS	PTN	
SLC1A5	MAP3K1	[16]
SNRPA	CDKN2A	
TUBB	CDKN2A/ TJP1/ MAP3K1	[27, 28]

In addition to the potential for combinatorial targeting of CDKN2A and MAP3K1, two over-expressed druggable proteins residing within this central component of the network were found to interact with more than one CIS-derived candidate cancer protein. CDK6 interacted with both CDKN2A and ZEB1, and as noted above, TUBB interacts with both MAP3K1 and CDKN2A. CDK6 and TUBB therefore act as network “bridging nodes”, that is, nodes that connect otherwise separate regions of the network and represent proteins through which previously identified candidate cancer genes driving MB development may exert common effects. Proteins with topological properties such as this represent likely points of cross-talk and are highly attractive therapeutic targets [29]. Whilst the above-mentioned analysis identified potential therapeutic targets common to all non-WNT MB, we also performed drug-target network analysis on individual molecular sub-groups of MB, identifying a list of novel drug candidates likely to target that specific subgroups of MB (Additional file 5: Fig. S2).

### Targeting microtubule dynamics induces significant regression of Gp3 and SHH patient-derived MB

TUBB emerged as the leading candidate based on its highly attractive network topology and pharmacogenomic properties. Epothilone B agents are a class of new generation microtubule-stabilizing agents (MSA) that bind to the β-tubulin subunit of microtubules. Of the three Epothilone B agents, patupilone, ixabepilone and sagupilone, ixabepilone is the most mature analogue in development, with published phase II and III data and regulatory approval for clinical use in the treatment of breast cancer [30]. On this basis, ixabepilone was selected for the targeting TUBB in this manuscript.

**Fig. 2:**
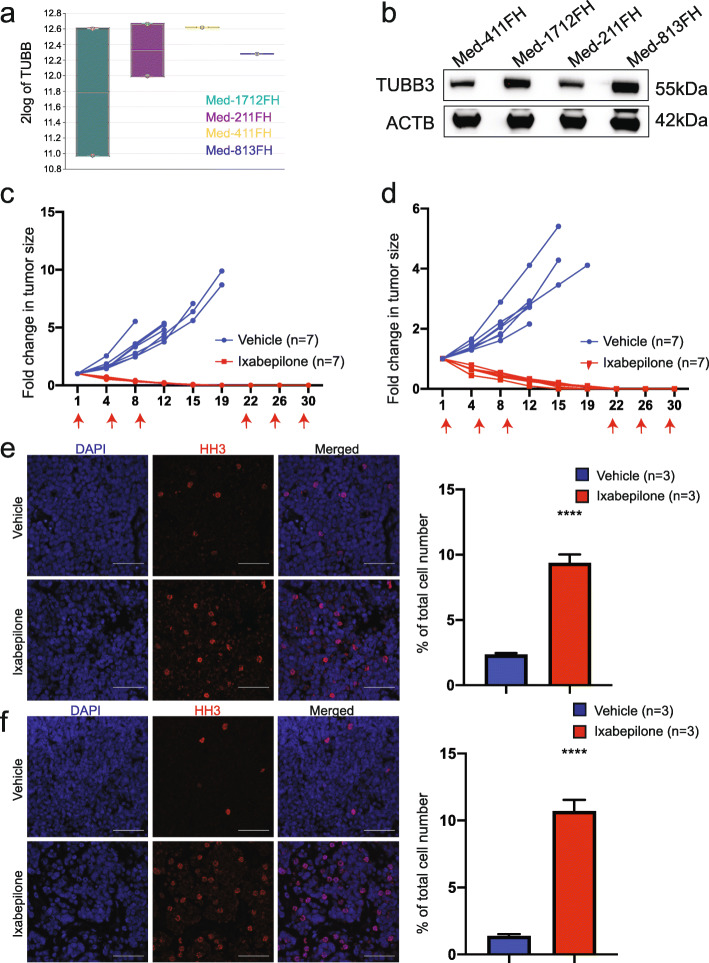
Ixabepilone causes regression of *MYC*-amplified Gp3 MB. **a** Relative mRNA expression of *TUBB* in Med-1712FH, Med-211FH, Med-411FH and Med-813FH PDX as compared to other paediatric brain tumours (PDX, matching human primary brain tumours and cell lines) using the R2 Genomics Analysis and Visualization Platform (http://r2.amc.nl). **b** Immunoblot analysis of TUBB3 and ACTB levels in Med-411FH, Med-1712FH, Med-211FH and Med-813FH PDX. Blots were scanned using BioRad ChemiDoc MP Imager and cropped using Adobe Illustrator. Fold change in tumour volume following treatment with ixabepilone or vehicle in mice bearing two Grp3 PDX models, **c** Med-211FH and **d** Med-411FH subcutaneous tumours. Tumour measurements were compared between vehicle and drug treatments using linear mixed models analysis in R. Treatment days are indicated by a red arrow on each graph. Representative images of HH3 immunostaining as a marker of mitosis respectively in Med-211FH (**e**, left) and Med-411FH (**f**, left) subcutaneous tumours following vehicle (top panel) and ixabepilone (bottom panel). Scale bar 20 μm. Quantitative analysis of HH3 staining in Med-211FH (**e**, right) and Med-411FH (**f**, right) tumours. Percentage of tumour cells staining positive were quantified for vehicle (n = 3) and drug-treated tumours (n = 3) using Image J software. Data are presented as the mean ± SEM. Statistical evaluation was performed using a Holm-Sidak corrected multiple t test with statistically significant differences indicated (*P < 0.05, **** *P < 0.0001*)

To investigate the anti-tumour efficacy of ixabepilone, we used well established PDX models of MB which are strictly maintained in vivo and shown to maintain the characteristics of the human primary tumours from which they were derived in terms of histology, immunohistochemistry, gene expression, DNA methylation, copy number and mutational profiles [25]. We analysed the results from previous microarray analyses of MB PDX models to evaluate expression of *TUBB* in our cohort [25]. High mRNA expression of *TUBB* was confirmed in a range of MB PDX including Med-1712FH (SHH), Med211FH (Gp3), Med-411FH (Gp3) and Med-813FH (SHH) (Fig. 2a), consistent with the network analysis of human MB (Fig. 1). Given tubulin expression is predominantly regulated post-transcriptionally and transcript levels do not necessarily reflect protein levels [31, 32], we defined tubulin protein levels in PDX lysates by western blot. Strong expression of beta isotype βIII (TUBB3) was confirmed in all MB PDX (Fig. 2b).

Med-211FH and Med-411FH PDX cells were implanted in the flank of mice and animals with established tumours were treated with vehicle or ixabepilone. Tumour growth was significantly reduced in drug-treated mice, with all Med-211FH (*P* < 0.001*,* Fig. 2c) and Med-411FH (*P* < 0.001*,* Fig. 2d) tumours exhibiting complete regression prior to completion of ixabepilone treatment, with no palpable tumour detected by day 19 and day 22 for Med-211FH and Med-411FH respectively. In contrast, growth continued at a high rate in the vehicle-treated tumours, increasing in size by approximately 5–6-fold for Med-211FH (Fig. 2c) and 5.62-fold for Med-411FH (Fig. 2d) before tumours reached maximum size requiring collection.

Whilst Med-211FH and Med-411FH are both models of Gp3 MB, our analyses predicted TUBB as a therapeutic candidate for multiple subgroups of MB, including SHH. On this basis, Med-1712FH PDX cells were implanted in the flank of mice and animals with established tumours were treated with vehicle or ixabepilone. Similar to the previous Gp3 PDX models, tumour growth was significantly reduced in drug-treated mice, with an average reduction in tumour volume of 67.61% (*P* < 0.001*,* Additional file 6: Fig. S3) whilst vehicle-treated tumours increased in size by approximately 2.47-fold. Together, these data serve as powerful validation of our strategy, demonstrating for the first time that the targeting of TUBB via microtubule stabilizer ixabepilone is an efficacious therapeutic approach for multiple subgroups of MB.

### In vivo anti-tumour activity of ixabepilone is associated with mitotic arrest, decreased cell proliferation and the induction of apoptosis

Ixabepilone binds to the β-tubulin subunit of microtubules, inducing microtubule polymerization and subsequent G2/M arrest, decreased proliferation and the induction of apoptosis [33–36]. On this basis, we sought to validate the mechanism of action and anti-tumour effects of Ixabepilone in vivo by immunofluorescence using phosphorylated histone H3 (pHH3) as a specific marker of mitosis, CC3 as a marker of apoptosis and Ki67 as a marker for proliferation. Med-211FH and Med-411FH PDX cells were implanted in the flank of mice and tumours were resected after just two drug administration doses. Ixabepilone demonstrated impressive single agent efficacy after 5 days (two doses), with an average reduction in tumour volume of 38.62% for Med-211FH (Fig. 2c) and 29.37% for Med-411FH (Fig. 2d).

**Fig. 3:**
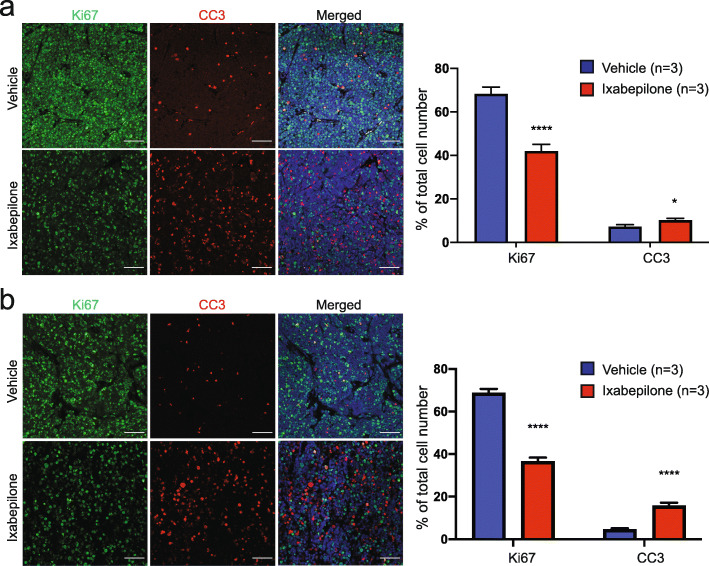
Ixabepilone induces apoptosis and decreases proliferation in *MYC*-amplified Gp3 MB. **a** Representative images of Ki67 and CC3 immunostaining as a marker of cell proliferation and apoptosis respectively in Med-211FH (**a**, left) and Med-411FH (**b**, left) subcutaneous tumours following vehicle (top panel) and ixabepilone (bottom panel). Scale bar 20 μm. Quantitative analysis of Ki67 and CC3 staining in Med-211FH (**a**, right) and Med-411FH (**b**, right) tumours. Percentage of tumour cells staining positive were quantified for vehicle (n = 3) and drug-treated tumours (n = 3) using Image J software. Data are presented as the mean ± SEM. Statistical evaluation was performed using a Holm-Sidak corrected multiple t test with statistically significant differences indicated (*P < 0.05, **** *P < 0.0001*)

Immunofluorescence analysis of drug-treated tumours showed a clear increase in the number of cells of drug-treated tumours in mitosis, with quantitative analysis indicating an increase of approximately 7% and 9% of pHH3 positive cells in drug-treated Med-211FH (*P* < 0.001, Fig. 2e) and Med-411FH (*P* < 0.001*,* Fig. 2f) tumours respectively. All drug-treated tumours of both Med-211FH and Med-411FH PDX also displayed decreased proliferation and increased apoptosis as compared to vehicle-treated tumours (Fig. 3). Quantitative analysis indicated drug-treated tumours displayed a reduction of approximately 26% and 32% of Ki67 positive cells in Med-211FH (*P* < 0.001*,* Fig. 3a right) and Med-411FH (*P* < 0.001, Fig. 3b right) respectively, with a concomitant increase of approximately 3% and 11% of CC3 positive cells in Med-211FH (*P* = 0. 013*,* Fig. 3a right) and Med-411FH (*P* < 0.001*,* Fig. 3b right) respectively. These data validate the hypothesis that the specific inhibition of microtubule dynamics via ixabepilone has significant in vivo anti-tumour effects in Gp3 PDX MB by arresting cells at mitosis, suppressing proliferation and inducing apoptosis.

### Ixabepilone treatment significantly extends the survival of mice engrafted with Gp3 and SHH MB PDXs

**Fig. 4:**
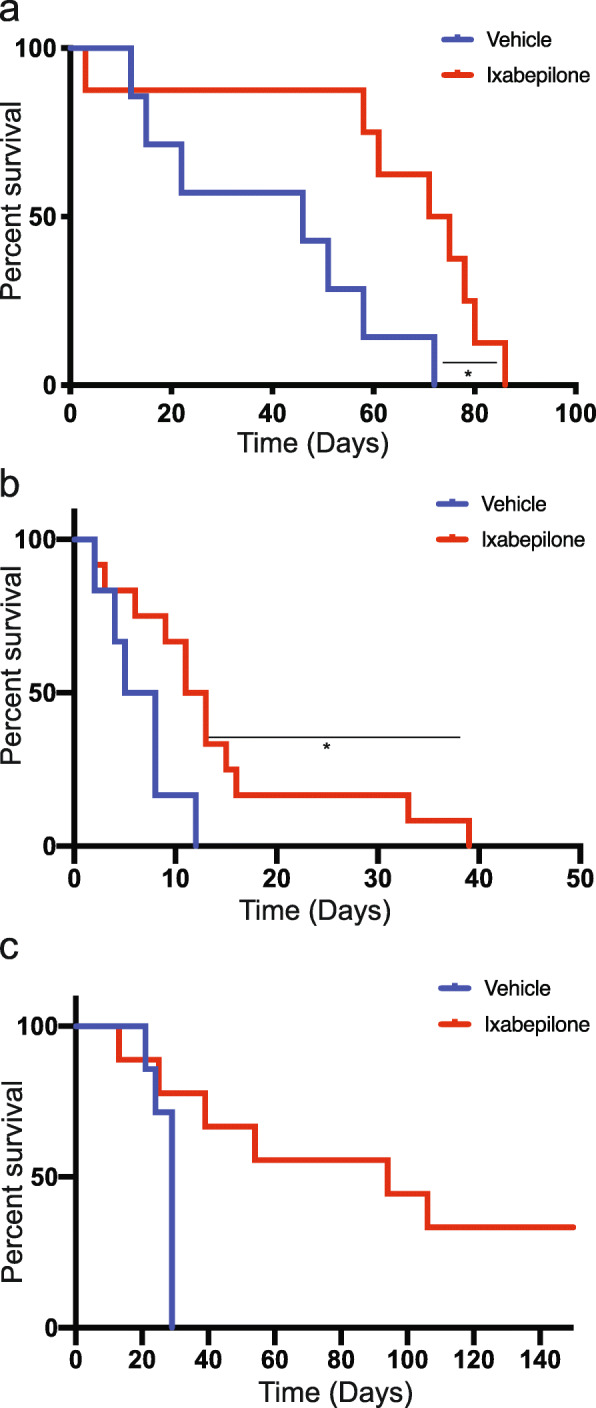
Ixabepilone significantly extends survival of mice bearing *MYC*-amplified Gp3 and SHH PDX. Kaplan-Meier curves representing survival of mice bearing MB PDX following treatment with ixabepilone or vehicle. **a** Med-211FH brain tumours treated with either ixabepilone (n = 8) or vehicle (n = 7), **b** Med-411FH brain tumours treated ixabepilone (n = 12) or vehicle (n = 7) and **c** BT084 brain tumours treated ixabepilone (n = 9) or vehicle (n = 7). Statistical evaluation was performed using the Log-rank (Mantel-Cox) test with statistically significant differences indicated (*P < 0.05)

We then investigated the efficacy of ixabepilone in the treatment of MYC-amplified PDX established as intracranial tumours in mice, previously demonstrated as the gold standard in the field of brain cancer modelling [37]. Mice bearing intracranial Med-211FH and Med-411FH tumours were enrolled and treated with ixabepilone or vehicle. Drug-treated animals showed a significant increase in survival for both Med-211FH (Fig. 4a, median survival = 72 days post treatment, 95% CI 60–infinity for treatment, 45 days post treatment 95% CI 14–infinity for vehicle, *P* = 0.016) and Med-411FH (Fig. 4b, median survival = 11 days post treatment 95% CI 8 to infinity for treatment vs. median survival = 5.5 days post treatment 95% CI 3–infinity for vehicle, *P* = 0.022). For Med-211FH, the probability of survival in the treatment group at 60 days post-treatment was 62.5% (95% CI 36.54–100) and 14.29% in the vehicle group (95% CI 2.33–87.69). For Med-411FH, the probability for survival in the treatment group at 30 days post-treatment was 25% (95% CI 9.38–66.61) and 0% in the vehicle group (95% CI 0–0).

**Fig. 5:**
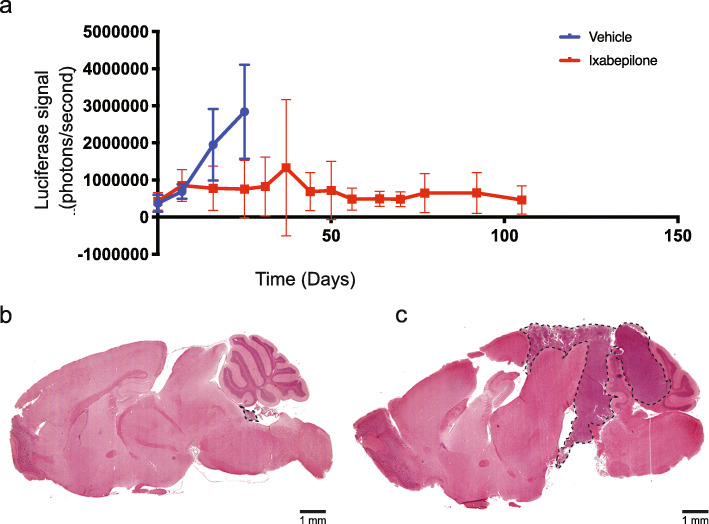
Ixabepilone causes dramatic reduction in tumour growth in orthotopic sonic hedgehog (SHH) medulloblastoma. **a** Tumour growth was monitored by bioluminescence following treatment with ixabepilone or vehicle in mice bearing BT084 SHH orthotopic tumours. Tumour measurements were compared between vehicle and drug treatments using linear mixed models analysis in R. Treatment days are indicated by vertical lines on the graph. Representative H and E images of ixabepilone-treated mice (**b**) at 171 and vehicle-treated mice (**c**) at 29 days post-treatment. Scale bar 1 mm

In a follow-up study, mice bearing orthotopic BT084 MB of the SHH subgroup (subtype alpha), which express a detectable signal of luciferase by bioluminescence, were treated with vehicle or ixabepilone. Drug-treated animals showed a significant increase in survival (Fig. 4c) where the probability for survival in the treatment group at 40 days post-treatment was 66.67% (95% CI 42–100 and 12.5% in the vehicle group (95% CI 2–78.19). Weekly evaluation of tumour growth by bioluminescence showed a significant reduction in tumour size in drug-treated animals as confirmed by the smaller increase in total photo flux from luciferase expressing BT084 xenografts (Fig. 5a).

Asymptomatic drug-treated mice bearing orthotopic BT084 MB were collected at 171 days post-treatment for histologic examination, with minimal tumour burden (Fig. 5b) observed compared to large tumours in vehicle-treated mice at 29 days (Fig. 5c). These results clearly demonstrate ixabepilone significantly prolonged survival of mice bearing intracranial tumours by dramatically reducing tumour size. Taken together, our findings confirm that the targeting of TUBB using a microtubule stabilizing agent is a viable therapeutic avenue for Group 3 and SHH MB, with these data representing biological validation of the predictive power of our systems pharmacogenomics approach to discover and validate novel drug candidates for all non-WNT MB.

## Discussion

Systems pharmacogenomics is a powerful approach for the discovery of novel druggable targets in cancer, with previous studies successfully using this approach in both in vitro and in vivo models to functionally predict and validate pharmacogenomic candidates [27, 28, 38–40]. Large scale genomic and transcriptomic analyses of MB have broadened our understanding of the molecular and genetic features driving MB development [2, 3, 5, 41].

Our increasing knowledge of these features has enabled the identification of a number of candidate genes as potential therapeutic targets for certain subgroups of this disease [18, 19, 42–44] and our own prior work confirmed a large number of genes functionally shown to drive tumorigenesis. However, methods to integrate the large amounts of data obtained from several mouse and human MB model analyses to the discovery and prioritization of novel drug targets for pre-clinical screening are still urgently required.

In this study, we have described a functional pharmacogenomics and network-driven systems biology approach based upon candidate cancer-promoting genes identified from our previously published *SB* mouse model of MB [16]. Whilst we could have expanded our network to include genes such as those identified in previous genomic studies [41, 45, 46], we chose to focus on the use of the CIS-derived cancer genes and the broader network to select for these novel therapeutic targets using the functional evidence of the mutagenesis screen as a guide. Many of these CIS-derived cancer genes are known to be mutated in human MB, as are other genes that interact with these in our network (Additional file 7: Table S4). The network itself is built using only experimentally verified PPI manually curated from the literature, and when combined with functional hits from the genetic screen, and expression data from patients, represents a powerful discovery tool. Integrating our functionally derived network with gene expression data from a large cohort of patient samples, we identify multiple proteins targeted by existing drugs that can be evaluated as potential MB therapeutics. This approach identified many confirmed targets with existing drugs that are currently used in the clinic or are in various stages of clinical trials in other cancers (for example clinical trials for drugs targeting AURKA and BRD4), emphasizing the efficacy of our approach [18, 19, 21, 47–50]. We previously validated CDK4/6 inhibition as a successful therapeutic approach for both Gp3 and SHH MB [20], consistent with an additional study demonstrating the efficacy of palbociclib as a single agent in the treatment of *MYC-*driven MB in an in vitro cerebellar human neural stem cell model of MB [51].

TUBB emerged as a leading candidate from our network analysis. Targeting TUBB via ixabepilone in four PDX models confirms this new generation MSA as an effective therapy for aggressive MB and validates an additional target from our functional network, demonstrating its power for the discovery of new MB therapies through drug repositioning. Significantly over-expressed druggable targets, such as TUBB, MYC, SMAD2, RARA and CDK6 for Gp3 MB, represent highly attractive therapeutic candidates due to their expression and centrality in the network, connected as they are to multiple driver genes [16]. Pharmacologic inhibition of these proteins has the potential to elicit both local and widespread global effects on the biological system sustaining MB, thereby stimulating a greater therapeutic response via simultaneous targeting of multiple biological pathways encompassed within these networks.

Microtubule targeting agents (MTAs) have been heavily exploited as anticancer agents, with certain classes of MTAs approved for several adult cancer types including breast, ovarian, lung, oesophageal, endometrial, cervical, prostrate and head and neck cancers [30, 52]. MTAs comprise many chemically diverse compounds which can be broadly classified into microtubule stabilizing agents, such as the taxanes and epothilones, and microtubule-destabilizing agents such as the *Vinca* alkaloids (reviewed in [49]). First-generation taxanes such as Paclitaxel and Docetaxel are high-affinity substrates for several ABC drug efflux transporters [50, 51]. Given the expression of these has been reported in a variety of central nervous system tumours including MB [52, 53], first-generation taxanes are expected to have limited efficacy in MB and were therefore not selected for this study.

Vincristine, a microtubule destabilizing agent specifically targeting TUBB and tubulin alpha-4A chain (TUBA4A), is a chemotherapeutic currently used for the management of standard-risk and high-risk MB [53]. However, due to its large size (MW 825 Daltons) and susceptibility to transport by ATP-binding cassette (ABC) drug efflux transporters such as P-glycoprotein (P-gp, encoded by the gene *MDR-*1/*ABCB1* gene) and multidrug resistance protein (MRP1, encoded by the *MRP1*/*ABCC1* gene) [54–56], vincristine does not penetrate well into brain tumour tissue. We have previously shown cabazitaxel, a second-generation taxane of the microtubule stabilizing class of MTAs, significantly extended survival of mice bearing orthotopic Med-211FH PDX tumours compared to vehicle controls [57]. Whilst these results were promising, growing clinical evidence indicates high expression of TUBB3 as a clinical biomarker of taxane resistance in various tumour types (reviewed in [32]), but has been shown to predict sensitivity to epothilone B compounds [58, 59]. Given the strong expression of TUBB3 in PDX models of MB observed in this study, epothilone B agents may be the preferred MTA and give rise to an overall more durable response. Semi-synthetic Epothilone B agent patupilone was previously found to reduce the proliferative capacity and clonogenicity in vitro of a variety of MB cell lines and delayed the growth of the D425 MB cell line in vivo following subcutaneous implantation [60]. The impressive efficacy of ixabepilone (Figs. 2, 4 and 5), an additional semi-synthetic second-generation Epothilone B compound, strongly highlights the potential of MSA for the treatment of children diagnosed with MB. Functional studies investigating the role of β-tubulin isotypes in sensitivity and resistance to epothilones in MB is required and is anticipated to facilitate the identification of biomarkers to accurately select MB patients most likely to respond to MTAs.

## Conclusions

The in vivo validation we present here for targeting TUBB with ixabepilone and previous validation of CDK6 targeting with palbociclib as two successful therapeutic options for MB support the power of our functionally derived network in identifying candidates and suggests that other players in this network with similar topological and biological properties may also have therapeutic efficacy in MB. Experimentally grounded molecular interaction networks constructed around functionally validated cancer driver genes capture real and significant biological mechanisms that are essential to cancer and can be productively targeted pharmacologically. By integrating extensive molecular profiling of patients as we have done here, such networks present powerful tools for the characterization of therapeutic candidates, the repositioning of drugs and ultimately the design of patient-informed therapies.

## Supplementary Information

**Additional file 1: Supplementary Figure 1.** Schematic of data integration to identify and validate therapeutic targets for MB.

**Additional file 2: Supplementary Table S1.** Network parameters for subgroup-specific network analysis.

**Additional file 3: Supplementary Table S2.** Putative drug targets and drugs in subgroup-specific networks.

**Additional file 4: Supplementary Table S3.** Expression Data associated with Figure 1.

**Additional file 5: Supplementary Figure S2.** Local protein interaction network representing significantly over-expressed druggable proteins for individual subgroups of MB.

**Additional file 6: Supplementary Figure S3.** Ixabepilone causes regression of sonic hedgehog (SHH) MB.

**Additional file 7: Supplementary Table S4.** Summary of mutational status in human Medulloblastoma of key CIS genes identified in the mutagenesis screen,

## Data Availability

All gene human gene expression data used in this study are available through the Gene Expression Ombibus at GSE37382. https://www.ncbi.nlm.nih.gov/geo/query/acc.cgi?acc=GSE37382 [41] and GSE167447. https://www.ncbi.nlm.nih.gov/geo/query/acc.cgi?acc=GSE167447 [61]. All Bioinformatics software used and cited in this study are open access and freely available.

## Abbreviations

MB: Medulloblastoma
CDK4: Cyclin-dependent kinase 4
CDK6: Cyclin-dependent kinase 6
TUBB: Tubulin beta chain
AURKA: Aurora kinase A
WNT: Wingless
CAD: Carbamoyl-phosphate synthetase 2, aspartate transcarbamylase, and dihydroorotase
SNPRA: Small nuclear ribonucleoprotein polypeptide A
SLC1A5: Solute carrier family 1 (neutral amino acid transporter) member 5
PTPRS: Protein tyrosine phosphatase receptor type S
P4HB: Protein disulfide-isomerase
CHEK2: Checkpoint kinase 2
SHH: Sonic hedgehog
Gp3: Group 3
Gp4: Group 4
SMO: Smoothened
PPI: Protein-protein interactions
SB: Sleeping Beauty
Ptch1: Patched 1
CIS: Common insertion sites
PDX: Patient-derived xenograft
CDKN2A: Cyclin-dependent kinase inhibitor 2A
ZEB1: Zinc finger E-box binding homeobox 1
TJP1: Tight junction protein 1
MAP3K1: Mitogen-activated protein kinase kinase kinase 1
FAM117B: Family with sequence similarity 117, member B
DYRK1A: Dual-specificity tyrosine-(Y)-phosphorylation regulated kinase 1A
UBE2D3: Ubiquitin-conjugating enzyme E2D3
UCHL1: Ubiquitin C-terminal hydrolase L1
RAB3C: Member RAS oncogene family
PTEN: Phosphatase and tensin homologue
MAGI1: Membrane-associated guanylate kinase, WW and PDZ domain
UBR5: Ubiquitin protein ligase E3 component N-recognin 5
MSA: Microtubule-stabilizing agent
TUBB3: Beta isotype βIII
pHH3: Phosphorylated histone H3
Ki67: Marker of proliferation Ki-67
CC3: Cleaved caspase 3
MYC: Myc proto-oncogene protein
CI: Confidence interval
MTAs: Microtubule targeting agents
TUBA4A: Tubulin alpha-4A chain
ABC: ATP-binding cassette
MRP1: Multidrug resistance protein
