# Supplementary material for: Systems pharmacogenomics identifies novel targets and clinically actionable therapeutics for medulloblastoma
Source: Genome Med. 2021 Jun 21;13:103. doi: 10.1186/s13073-021-00920-z (PMC8215804; doi:10.1186/s13073-021-00920-z)
Supplement: Supplementary file 1 — Additional file 1: Supplementary Figure 1. Schematic of data integration to identify and validate therapeutic targets for MB. [file 13073_2021_920_MOESM1_ESM.pdf]

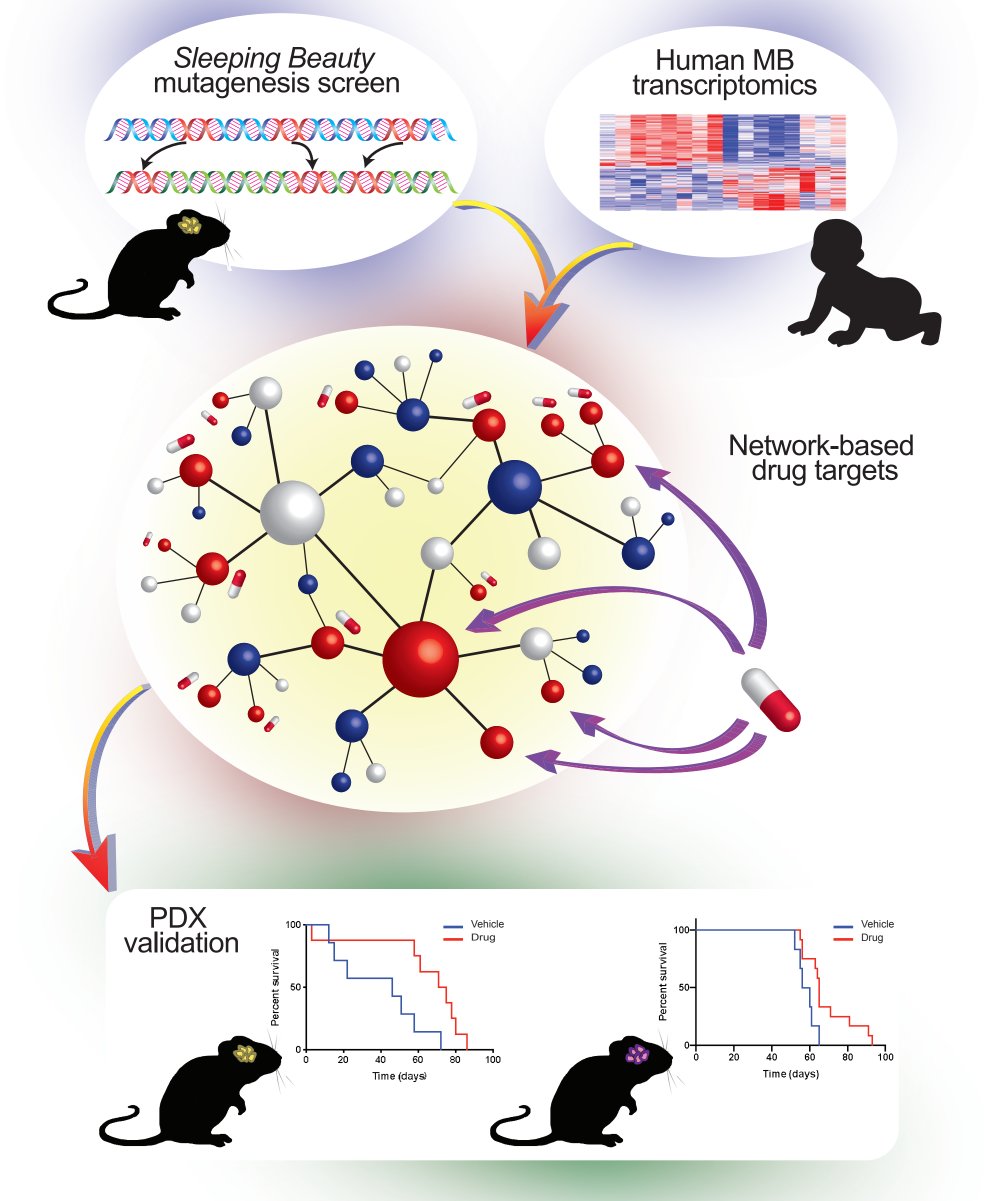

Supplementary Figure S1: SB-induced mutagenesis was performed in the Ptch1 heterozygous mouse model and transposon CIS were identified. The local protein network for each CIS-derived candidate cancer gene was generated from experimentally determined protein interaction data and these local protein networks integrated to generate a PPI network comprising the CISs and their interacting proteins. Human MB transcriptomics data was used to identify differentially expressed genes common to all non-WNT MB, which was used to refine our protein interaction network to include only over-expressed genes and their directly interacting proteins. Drugs and their molecular targets were mapped to this network, with subsequent validation of these novel druggable proteins in PDX models of MB
