# Supplementary material for: Systems pharmacogenomics identifies novel targets and clinically actionable therapeutics for medulloblastoma
Source: Genome Med. 2021 Jun 21;13:103. doi: 10.1186/s13073-021-00920-z (PMC8215804; doi:10.1186/s13073-021-00920-z)
Supplement: Supplementary file 5 — Additional file 5: Supplementary Figure S2. Local protein interaction network representing significantly over-expressed druggable proteins for individual subgroups of MB. [file 13073_2021_920_MOESM5_ESM.pdf]

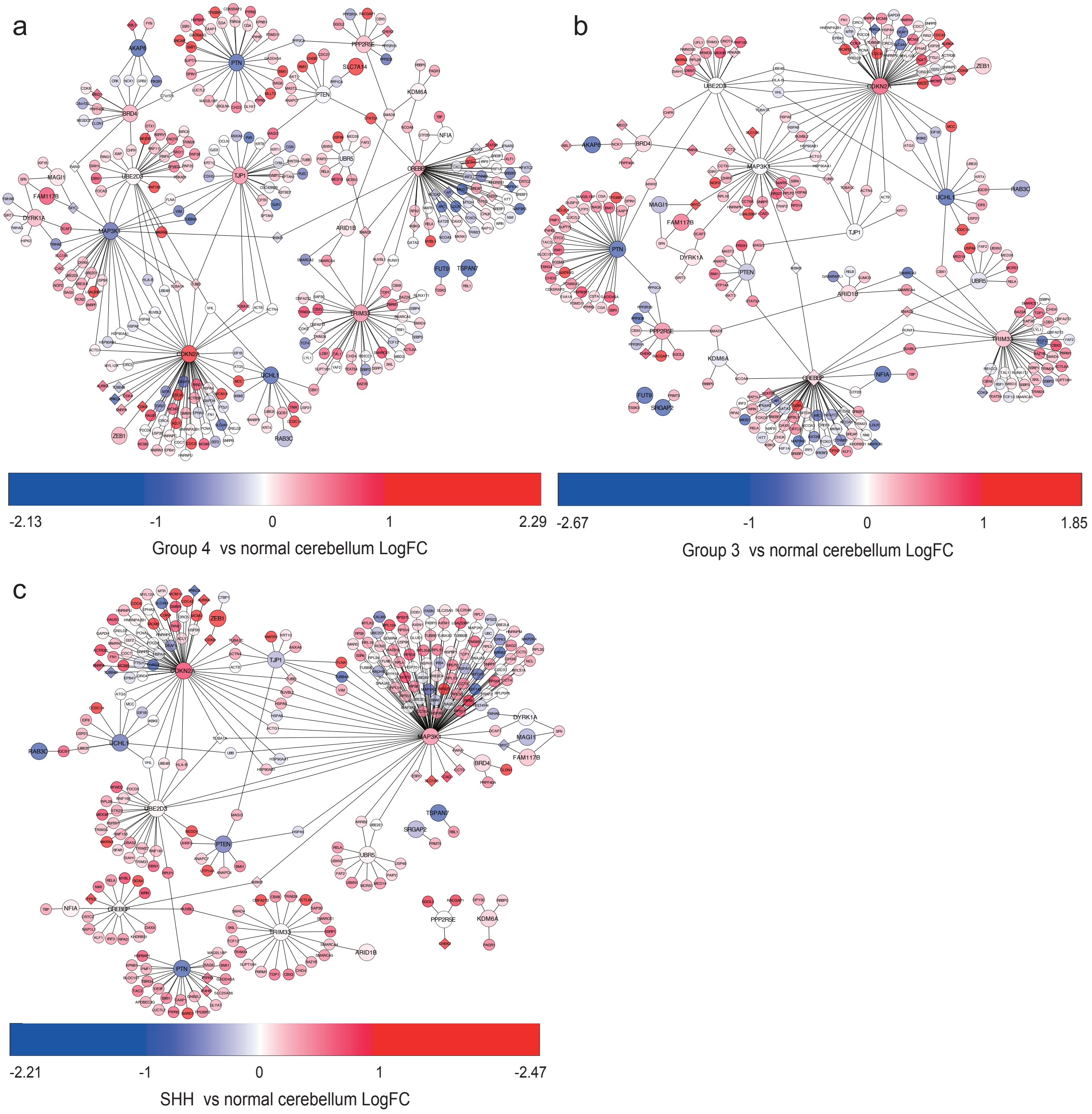

Supplementary Figure S2: Local protein interaction network representing the significantly over-expressed druggable proteins for (a) Group 4, (b) Group 3 and (c) SHH MB. CIS-derived candidate cancer genes are represented by large circles, whilst small circles represent the proteins that interact with these CI-derived cancdidates. Nodes coloured in red-light red represent significantly over-expressed proteins, whilst blue-light blue represent significantly under-expressed proteins of that respective subgroup.
