# Supplementary material for: Systems pharmacogenomics identifies novel targets and clinically actionable therapeutics for medulloblastoma
Source: Genome Med. 2021 Jun 21;13:103. doi: 10.1186/s13073-021-00920-z (PMC8215804; doi:10.1186/s13073-021-00920-z)
Supplement: Supplementary file 6 — Additional file 6: Supplementary Figure S3. Ixabepilone causes regression of sonic hedgehog (SHH) MB. [file 13073_2021_920_MOESM6_ESM.pdf]

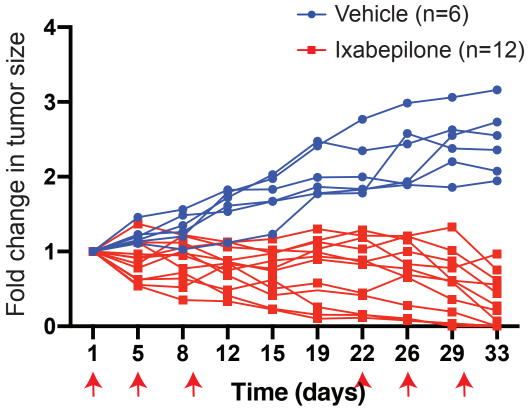

Supplementary Figure S3: Ixabepilone causes regression of sonic hedgehog (SHH) MB. Fold change in tumour volume following treatment with ixabepilone or vehicle in mice bearing Med-1712FH SHH subcutaneous tumours. Tumour measurements were compared between vehicle and drug treatments using linear mixed models analysis in R. Treatment days are indicated by a red arrow on each graph.
